# Quinolinium 8-hy­droxy-7-iodo­quinoline-5-sulfonate 0.8-hydrate

**DOI:** 10.1107/S1600536812046247

**Published:** 2012-11-14

**Authors:** Graham Smith

**Affiliations:** a Science and Engineering Faculty, Queensland University of Technology, GPO Box 2434, Brisbane, 4001, Australia

## Abstract

In the crystal structure of the title hydrated quinolinium salt of ferron (8-hy­droxy-7-iodo­quinoline-5-sulfonic acid), C_9_H_7_N^+^·C_9_H_5_INO_4_S^−^·0.8H_2_O, the quinolinium cation is fully disordered over two sites (occupancy factors fixed at 0.63 and 0.37) lying essentially within a common plane and with the ferron anions forming π–π-associated stacks down the *b* axis [minimum ring centroid separation = 3.462 (6) Å]. The cations and anions are linked into chains extending along *c* through hy­droxy O—H⋯O and quinolinium N—H⋯O hydrogen bonds to sulfonate O-atom acceptors which are also involved in water O—H⋯O hydrogen-bonding inter­actions along *b*, giving a two-dimensional network.

## Related literature

For the crystal structure of ferron, see: Balasubramanian & Muthiah (1996 ▶). For analytical applications of ferron, see: Vogel (1964 ▶). For the crystal structures of other non-zwitterionic compounds of ferron, see: Hemamalini *et al.* (2004 ▶); Smith *et al.* (2004 ▶, 2007 ▶).
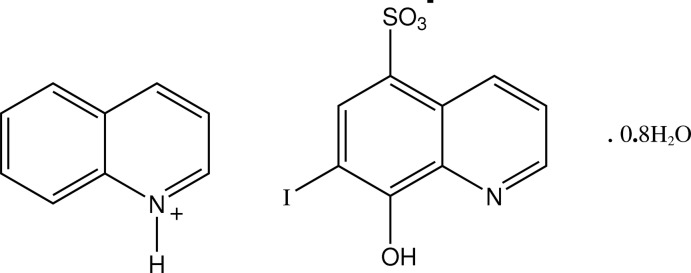


## Experimental

### 

#### Crystal data

C_9_H_8_N^+^·C_9_H_5_INO_4_S^−^·0.8H_2_O
*M* *_r_* = 494.69
Orthorhombic, 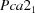

*a* = 16.2403 (5) Å
*b* = 7.1539 (3) Å
*c* = 15.2458 (5) Å
*V* = 1771.28 (11) Å^3^
*Z* = 4
Mo *K*α radiation
μ = 1.96 mm^−1^
*T* = 200 K
0.32 × 0.25 × 0.12 mm

#### Data collection

Oxford Diffraction Gemini-S CCD-detector diffractometer
Absorption correction: multi-scan (*CrysAlis PRO*; Agilent, 2012 ▶) *T*_min_ = 0.906, *T*_max_ = 0.980
6143 measured reflections
3207 independent reflections
2709 reflections with *I* > 2σ(*I*)
*R*_int_ = 0.028

#### Refinement

*R*[*F* ^2^ > 2σ(*F* ^2^)] = 0.040
*wR*(*F* ^2^) = 0.082
*S* = 1.18
3207 reflections
244 parameters
1 restraint
H-atom parameters constrained
Δρ_max_ = 0.65 e Å^−3^
Δρ_min_ = −0.66 e Å^−3^
Absolute structure: Flack (1983 ▶), 789 Friedel pairs
Flack parameter: 0.01 (3)

### 

Data collection: *CrysAlis PRO* (Agilent, 2012 ▶); cell refinement: *CrysAlis PRO*; data reduction: *CrysAlis PRO*; program(s) used to solve structure: *SIR92* (Altomare *et al.*, 1993 ▶); program(s) used to refine structure: *SHELXL97* (Sheldrick, 2008 ▶) within *WinGX* (Farrugia, 2012 ▶); molecular graphics: *PLATON* (Spek, 2009 ▶); software used to prepare material for publication: *PLATON*.

## Supplementary Material

Crystal structure: contains datablock(s) I, global. DOI: 10.1107/S1600536812046247/su2523sup1.cif

Structure factors: contains datablock(s) I. DOI: 10.1107/S1600536812046247/su2523Isup2.hkl

Supplementary material file. DOI: 10.1107/S1600536812046247/su2523Isup3.cml

Additional supplementary materials:  crystallographic information; 3D view; checkCIF report

## Figures and Tables

**Table 1:** Hydrogen-bond geometry (Å, °)

*D*—H⋯*A*	*D*—H	H⋯*A*	*D*⋯*A*	*D*—H⋯*A*
N1*A*—H1*A*⋯O53^i^	0.86	1.97	2.783 (10)	157
N1*B*—H1*B*⋯O53^i^	0.86	1.88	2.725 (16)	166
O8—H8⋯O52^ii^	0.81	2.13	2.769 (7)	135
O1*W*—H11*W*⋯O52	0.89	2.18	3.066 (9)	179
O1*W*—H12*W*⋯O51^iii^	0.90	2.18	3.080 (8)	178

Symmetry codes: (i) 
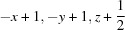
; (ii) 
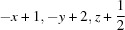
; (iii) 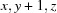.

**Fig. 1.**
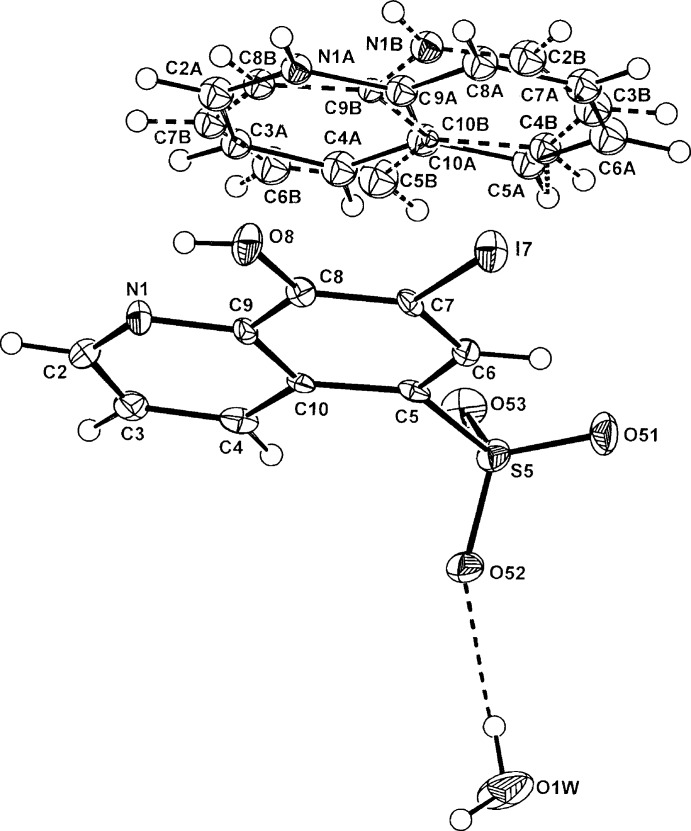
Molecular configuration and atom naming for the individual cation, the disordered anion components (A and B) and the water species in the title compound. The minor-occupancy B anion is shown with broken bonds and displacement ellipsoids are drawn at the 30% probability level. The intra- and inter-species hydrogen bonds are shown as a dashed lines.

**Fig. 2.**
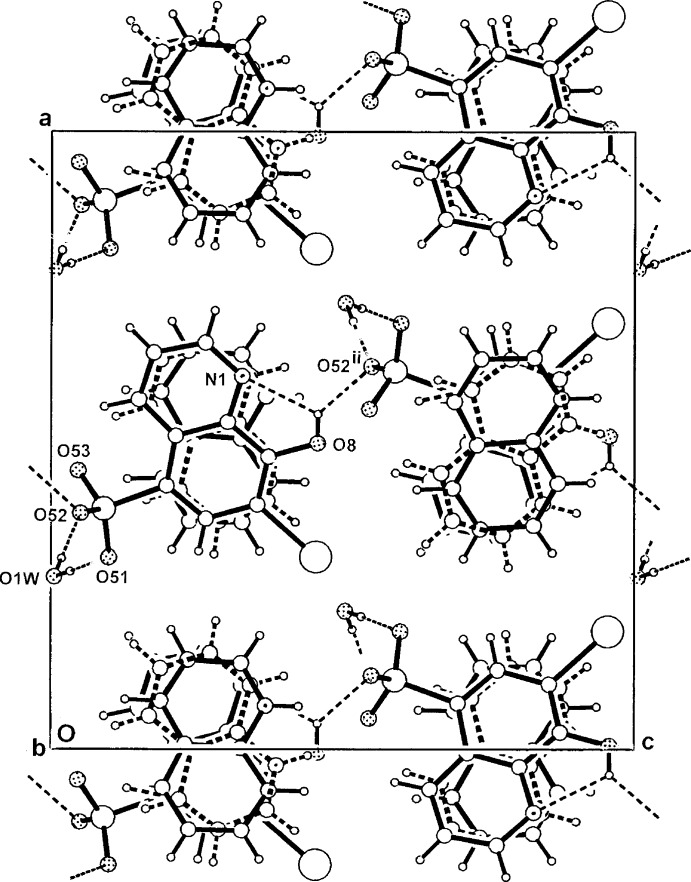
The stacking of the cation and anion rings down the b axis in the crystal of the title compound.

**Fig. 3.**
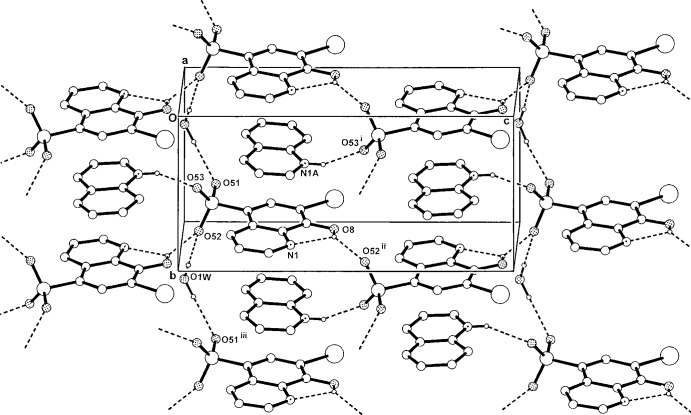
A perspective view of the crystal packing of the title compound viewed along the a axis, showing the inter-chain water hydrogen-bonding associations (dashed lines; see Table for details; symmetry codes: (i) -x+1, -y+1, z+1/2; (ii) -x+1, -y+2, z+1/2; (iii) x, y+1, z).

## supplementary crystallographic information

### Comment

Ferron (8-hydroxy-7-iodoquinoline-5-sulfonic acid) is a bidentate complexing agent which has analytical applications as a selective colour reagent for the detection of iron(III) but not iron(II) (Vogel, 1964). The crystal structure of ferron (Balasubramanian & Muthiah, 1996) has shown that the molecule exists as a sulfonate-quinolinium zwitterion. As a sulfonic acid, ferron is potentially capable of protonating most Lewis bases, but the crystal structures of only a small number of such salts have been reported. With 8-hydroxyquinoline, a 1:1 sesquihydrate is formed (Smith *et al.*, 2004) and with bifunctional 4,4^'^-bipyridine (Hemamalini *et al.*, 2004) a monoprotonated 1:1 dihydrate is found. A common structural feature in these ferron proton-transfer salts is the presence of *R*^2^_2_(10) cyclic hydrogen-bonded ferron···ferron dimers involving the 8-hydroxy donor and hetero-N acceptor groups. Reaction of ferron with quinoline gave the title chemically stable 1:1 hydrated salt, whose crystal structure is reported on herein.

In the title compound, Fig. 1, the quinolinium cation is fully disordered over two sites *A* and *B* with occupancy factors fixed at 0.63 and 0.37, lying essentially within a common plane. These cations are linked to the anions through both quinolinium N—H···O and hydroxyl O—H···O and hydrogen bonds to sulfonate O-atom acceptors (Table 1), forming chains extending along *c*. Water O—H···O_sulfonate_ hydrogen-bonding interactions together with cation–anion ring π–π associations [minimum ring centroid separation = 3.462 (6) Å] link the chains down the *b* axial direction, giving a two-dimensional network structure (Figs. 2 and 3). The ferron–ferron dimeric association is not present. In the crystal, there are relatively short intra-anionic I7···O51^iv^ interactions [3.027 (5) Å] [symmetry code (iv): *x* + 1/2,-*y*, *z*].

With the ferron anion, the short intra-anionic O8—H8···N1 association [2.693 (7) Å] is present, similar to that found in other non-zwitterionic compounds of ferron (Hemamalini *et al.*, 2004; Smith *et al.*, 2004, 2007). Also the common aromatic ring C6–H6···O51_sulfonate_ association [2.827 (8) Å] maintains the S5–O51 bond close to the extended plane of the aromatic ring [torsion angle C10—C5—S5—O51, 171.1 (5) °].

### Experimental

The title compound was synthesized by heating a solution containing 1 mmol of 8-hydroxy-7-iodoquinoline-5-sulfonic acid (ferron) and 1 mmol of quinoline in 50 ml of 50% ethanol-water for 10 min under reflux. After concentration to *ca*. 40 ml, partial room temperature evaporation of the hot-filtered solution gave yellow flat prisms of the title compound (m.p. 460.6–462.3 K) from which a specimen was cleaved for the X-ray analysis.

### Refinement

Hydrogen atoms on the water molecule and the hydroxyl group were located in a difference-Fourier synthesis but were subsequently allowed to ride in the refinement with *U*_iso_(H) = 1.5*U*_eq_(O). Other H-atoms were included in the refinement in calculated positions with N—H = 0.86 Å or C—H = 0.93 Å and were also treated as riding, with *U*_iso_(H) = 1.2*U*_eq_(C). The site occupancy of the water molecule was determined as 0.801 (12) and was subsequently fixed as 0.80. The quinolinium cation was completely disordered laterally within a common plane and the minor component (*B*) was subsequently located and its occupancy determined as 0.373 (14). Because of the instability in the anisotropic displacement parameters for both components, these were refined isotropically. The maximum difference peak was 0.64 e Å^-3^ 1.07 Å from I7.

### Crystal data

**Table:**

C_9_H_8_N^+^·C_9_H_5_INO_4_S^−^·0.8H_2_O	*F*(000) = 976
*M**_r_* = 494.69	*D*_x_ = 1.855 Mg m^−^^3^
Orthorhombic, *P**c**a*2_1_	Mo *K*α radiation, λ = 0.71073 Å
Hall symbol: P 2c -2ac	Cell parameters from 1806 reflections
*a* = 16.2403 (5) Å	θ = 3.4–28.9°
*b* = 7.1539 (3) Å	µ = 1.96 mm^−^^1^
*c* = 15.2458 (5) Å	*T* = 200 K
*V* = 1771.28 (11) Å^3^	Flat prism, yellow
*Z* = 4	0.32 × 0.25 × 0.12 mm

### Data collection

**Table:**

Oxford Diffraction Gemini-S CCD-detector diffractometer	3207 independent reflections
Radiation source: fine-focus sealed tube	2709 reflections with *I* > 2σ(*I*)
Graphite monochromator	*R*_int_ = 0.028
Detector resolution: 16.077 pixels mm^-1^	θ_max_ = 28.9°, θ_min_ = 3.4°
ω scans	*h* = −22→15
Absorption correction: multi-scan (*CrysAlis PRO*; Agilent, 2012)	*k* = −6→9
*T*_min_ = 0.906, *T*_max_ = 0.980	*l* = −19→19
6143 measured reflections	

### Refinement

**Table:**

Refinement on *F*^2^	Secondary atom site location: difference Fourier map
Least-squares matrix: full	Hydrogen site location: inferred from neighbouring sites
*R*[*F*^2^ > 2σ(*F*^2^)] = 0.040	H-atom parameters constrained
*wR*(*F*^2^) = 0.082	*w* = 1/[σ^2^(*F*_o_^2^) + (0.0181*P*)^2^ + 3.2291*P*] where *P* = (*F*_o_^2^ + 2*F*_c_^2^)/3
*S* = 1.18	(Δ/σ)_max_ = 0.004
3207 reflections	Δρ_max_ = 0.65 e Å^−^^3^
244 parameters	Δρ_min_ = −0.66 e Å^−^^3^
1 restraint	Absolute structure: Flack (1983), 789 Friedel pairs
Primary atom site location: structure-invariant direct methods	Flack parameter: 0.01 (3)

### Special details

**Table:**

Geometry. Bond distances, angles *etc*. have been calculated using the rounded fractional coordinates. All su's are estimated from the variances of the (full) variance-covariance matrix. The cell e.s.d.'s are taken into account in the estimation of distances, angles and torsion angles
Refinement. Refinement of *F*^2^ against ALL reflections. The weighted *R*-factor *wR* and goodness of fit *S* are based on *F*^2^, conventional *R*-factors *R* are based on *F*, with *F* set to zero for negative *F*^2^. The threshold expression of *F*^2^ > σ(*F*^2^) is used only for calculating *R*-factors(gt) *etc*. and is not relevant to the choice of reflections for refinement. *R*-factors based on *F*^2^ are statistically about twice as large as those based on *F*, and *R*- factors based on ALL data will be even larger.

### Fractional atomic coordinates and isotropic or equivalent isotropic displacement parameters (Å^2^)

**Table:**

	*x*	*y*	*z*	*U*_iso_*/*U*_eq_	Occ. (<1)
N1A	0.5706 (6)	0.4412 (12)	0.3693 (6)	0.020 (2)*	0.630
C2A	0.6389 (9)	0.4939 (16)	0.3279 (9)	0.025 (3)*	0.630
C3A	0.6447 (7)	0.4807 (15)	0.2359 (9)	0.019 (2)*	0.630
C4A	0.5784 (8)	0.4161 (17)	0.1888 (8)	0.026 (3)*	0.630
C5A	0.4375 (7)	0.2937 (15)	0.1920 (9)	0.023 (3)*	0.630
C6A	0.3681 (8)	0.2443 (17)	0.2377 (9)	0.030 (2)*	0.630
C7A	0.3664 (8)	0.2600 (17)	0.3288 (10)	0.026 (2)*	0.630
C8A	0.4325 (10)	0.3261 (17)	0.3750 (8)	0.026 (3)*	0.630
C9A	0.5015 (8)	0.3760 (17)	0.3272 (8)	0.021 (3)*	0.630
C10A	0.5084 (11)	0.356 (3)	0.2307 (12)	0.022 (5)*	0.630
C8B	0.6037 (15)	0.466 (3)	0.3434 (14)	0.020 (4)*	0.370
C9B	0.5273 (13)	0.388 (3)	0.3220 (12)	0.013 (4)*	0.370
C10B	0.4986 (15)	0.362 (3)	0.2426 (16)	0.006 (6)*	0.370
C3B	0.3682 (13)	0.254 (3)	0.2889 (17)	0.029 (4)*	0.370
C4B	0.4180 (12)	0.288 (3)	0.2210 (14)	0.020 (4)*	0.370
C5B	0.5542 (14)	0.390 (3)	0.1688 (15)	0.032 (5)*	0.370
C6B	0.6308 (13)	0.451 (3)	0.1885 (15)	0.030 (4)*	0.370
C7B	0.6552 (12)	0.496 (3)	0.2747 (15)	0.022 (4)*	0.370
N1B	0.4736 (11)	0.341 (2)	0.3879 (10)	0.022 (3)*	0.370
C2B	0.4005 (15)	0.285 (3)	0.3719 (14)	0.028 (5)*	0.370
I7	0.30996 (2)	0.75195 (7)	0.45473 (4)	0.0259 (1)	
S5	0.38785 (10)	0.7547 (3)	0.09027 (10)	0.0232 (4)	
O8	0.4943 (2)	0.8886 (6)	0.4574 (4)	0.0265 (11)	
O51	0.3101 (3)	0.6580 (7)	0.0999 (3)	0.0327 (16)	
O52	0.3807 (3)	0.9388 (7)	0.0506 (3)	0.0280 (16)	
O53	0.4497 (3)	0.6391 (7)	0.0466 (3)	0.0323 (16)	
N1	0.6037 (3)	0.9565 (7)	0.3285 (4)	0.0220 (17)	
C2	0.6580 (4)	0.9911 (9)	0.2656 (5)	0.027 (2)	
C3	0.6418 (4)	0.9684 (9)	0.1760 (5)	0.0233 (19)	
C4	0.5659 (4)	0.9043 (9)	0.1505 (4)	0.0223 (19)	
C5	0.4255 (4)	0.7936 (7)	0.1981 (4)	0.0150 (17)	
C6	0.3719 (4)	0.7600 (9)	0.2665 (4)	0.0183 (17)	
C7	0.3948 (4)	0.7926 (7)	0.3545 (4)	0.0147 (17)	
C8	0.4722 (4)	0.8578 (8)	0.3737 (4)	0.0173 (17)	
C9	0.5289 (4)	0.8920 (8)	0.3042 (4)	0.0157 (17)	
C10	0.5063 (4)	0.8627 (8)	0.2143 (4)	0.0167 (17)	
O1W	0.2775 (5)	1.2846 (9)	0.0055 (5)	0.057 (3)	0.800
H4A	0.58090	0.41330	0.12790	0.0310*	0.630
H5A	0.43630	0.28440	0.13110	0.0270*	0.630
H6A	0.32210	0.20010	0.20780	0.0360*	0.630
H7A	0.31910	0.22470	0.35900	0.0310*	0.630
H8A	0.43110	0.33690	0.43570	0.0310*	0.630
H1A	0.56960	0.44830	0.42560	0.0240*	0.630
H2A	0.68320	0.53980	0.35990	0.0300*	0.630
H3A	0.69290	0.51530	0.20730	0.0220*	0.630
H1B	0.48960	0.34940	0.44150	0.0260*	0.370
H2B	0.36610	0.26310	0.41960	0.0330*	0.370
H3B	0.31450	0.21280	0.28100	0.0350*	0.370
H4B	0.40210	0.26440	0.16340	0.0240*	0.370
H5B	0.53800	0.36630	0.11140	0.0380*	0.370
H6B	0.66890	0.46380	0.14340	0.0360*	0.370
H7B	0.70690	0.54790	0.28460	0.0270*	0.370
H8B	0.61840	0.49450	0.40080	0.0250*	0.370
H2	0.71010	1.03290	0.28190	0.0320*	
H3	0.68190	0.99650	0.13450	0.0280*	
H4	0.55400	0.88840	0.09130	0.0270*	
H6	0.31940	0.71470	0.25440	0.0220*	
H8	0.54190	0.92330	0.45790	0.0390*	
H11W	0.30700	1.18390	0.01810	0.0850*	0.800
H12W	0.28600	1.39490	0.03310	0.0850*	0.800

### Atomic displacement parameters (Å^2^)

**Table:**

	*U*^11^	*U*^22^	*U*^33^	*U*^12^	*U*^13^	*U*^23^
I7	0.0229 (2)	0.0360 (2)	0.0189 (2)	−0.0052 (2)	0.0053 (2)	−0.0028 (2)
S5	0.0250 (8)	0.0290 (8)	0.0156 (6)	−0.0017 (8)	−0.0034 (6)	0.0023 (7)
O8	0.025 (2)	0.036 (2)	0.0184 (18)	−0.0057 (18)	−0.006 (3)	−0.007 (3)
O51	0.030 (3)	0.043 (3)	0.025 (2)	−0.017 (2)	−0.010 (2)	0.003 (2)
O52	0.028 (3)	0.031 (3)	0.025 (2)	0.000 (2)	−0.003 (2)	0.010 (2)
O53	0.045 (3)	0.031 (3)	0.021 (2)	0.006 (3)	0.000 (2)	−0.009 (2)
N1	0.017 (3)	0.022 (3)	0.027 (3)	−0.005 (2)	−0.003 (2)	−0.001 (3)
C2	0.018 (3)	0.023 (4)	0.041 (4)	0.002 (3)	−0.007 (3)	0.007 (3)
C3	0.018 (3)	0.017 (3)	0.035 (4)	0.006 (3)	0.005 (3)	0.003 (3)
C4	0.028 (4)	0.016 (3)	0.023 (3)	0.005 (3)	0.004 (3)	0.004 (3)
C5	0.025 (3)	0.008 (3)	0.012 (3)	0.000 (2)	0.001 (2)	0.000 (2)
C6	0.018 (3)	0.019 (3)	0.018 (3)	−0.003 (3)	−0.002 (2)	0.004 (3)
C7	0.013 (3)	0.011 (3)	0.020 (3)	−0.001 (2)	0.006 (2)	0.001 (2)
C8	0.020 (3)	0.014 (3)	0.018 (3)	0.001 (2)	0.001 (3)	−0.004 (3)
C9	0.013 (3)	0.009 (3)	0.025 (3)	0.000 (2)	0.001 (2)	−0.001 (2)
C10	0.018 (3)	0.012 (3)	0.020 (3)	−0.001 (2)	0.000 (3)	0.008 (3)
O1W	0.070 (5)	0.027 (4)	0.073 (5)	0.023 (4)	−0.034 (4)	−0.010 (4)

### Geometric parameters (Å, º)

**Table:**

I7—C7	2.078 (6)	C8A—C9A	1.38 (2)
S5—O52	1.454 (5)	C8B—C9B	1.40 (3)
S5—O51	1.447 (5)	C9A—C10A	1.48 (2)
S5—O53	1.462 (5)	C9B—C10B	1.31 (3)
S5—C5	1.776 (6)	C2A—H2A	0.9300
O8—C8	1.344 (8)	C2B—H2B	0.9300
O8—H8	0.8100	C3A—H3A	0.9300
O1W—H12W	0.9000	C3B—H3B	0.9300
O1W—H11W	0.8900	C4A—H4A	0.9300
N1A—C2A	1.331 (17)	C4B—H4B	0.9300
N1A—C9A	1.374 (16)	C5A—H5A	0.9300
N1B—C9B	1.37 (3)	C5B—H5B	0.9300
N1B—C2B	1.28 (3)	C6A—H6A	0.9300
N1A—H1A	0.8600	C6B—H6B	0.9300
N1B—H1B	0.8600	C7A—H7A	0.9300
N1—C2	1.326 (9)	C7B—H7B	0.9300
N1—C9	1.351 (8)	C8A—H8A	0.9300
C2A—C3A	1.409 (19)	C8B—H8B	0.9300
C2B—C3B	1.39 (3)	C2—C3	1.401 (11)
C3A—C4A	1.374 (17)	C3—C4	1.371 (9)
C3B—C4B	1.34 (3)	C4—C10	1.404 (9)
C4A—C10A	1.37 (2)	C5—C6	1.380 (9)
C4B—C10B	1.45 (3)	C5—C10	1.424 (9)
C5A—C10A	1.37 (2)	C6—C7	1.412 (9)
C5A—C6A	1.371 (18)	C7—C8	1.372 (9)
C5B—C6B	1.35 (3)	C8—C9	1.425 (9)
C5B—C10B	1.46 (3)	C9—C10	1.434 (9)
C6A—C7A	1.39 (2)	C2—H2	0.9300
C6B—C7B	1.41 (3)	C3—H3	0.9300
C7A—C8A	1.37 (2)	C4—H4	0.9300
C7B—C8B	1.36 (3)	C6—H6	0.9300
			
O51—S5—O52	113.9 (3)	C4B—C3B—H3B	122.00
O51—S5—O53	112.1 (3)	C10A—C4A—H4A	120.00
O51—S5—C5	106.3 (3)	C3A—C4A—H4A	120.00
O52—S5—O53	112.2 (3)	C3B—C4B—H4B	122.00
O52—S5—C5	105.7 (3)	C10B—C4B—H4B	122.00
O53—S5—C5	105.9 (3)	C10A—C5A—H5A	118.00
C8—O8—H8	108.00	C6A—C5A—H5A	118.00
H11W—O1W—H12W	122.00	C10B—C5B—H5B	122.00
C2A—N1A—C9A	123.7 (10)	C6B—C5B—H5B	122.00
C2B—N1B—C9B	121.9 (17)	C5A—C6A—H6A	120.00
C9A—N1A—H1A	118.00	C7A—C6A—H6A	120.00
C2A—N1A—H1A	118.00	C5B—C6B—H6B	119.00
C9B—N1B—H1B	119.00	C7B—C6B—H6B	119.00
C2B—N1B—H1B	119.00	C8A—C7A—H7A	119.00
C2—N1—C9	117.6 (6)	C6A—C7A—H7A	119.00
N1A—C2A—C3A	120.6 (12)	C8B—C7B—H7B	120.00
N1B—C2B—C3B	125 (2)	C6B—C7B—H7B	120.00
C2A—C3A—C4A	119.4 (11)	C7A—C8A—H8A	121.00
C2B—C3B—C4B	117 (2)	C9A—C8A—H8A	122.00
C3A—C4A—C10A	120.7 (13)	C9B—C8B—H8B	122.00
C3B—C4B—C10B	116 (2)	C7B—C8B—H8B	122.00
C6A—C5A—C10A	123.8 (14)	N1—C2—C3	124.0 (6)
C6B—C5B—C10B	116 (2)	C2—C3—C4	119.0 (6)
C5A—C6A—C7A	120.1 (12)	C3—C4—C10	119.6 (6)
C5B—C6B—C7B	123 (2)	C6—C5—C10	120.7 (6)
C6A—C7A—C8A	121.7 (12)	S5—C5—C6	117.1 (5)
C6B—C7B—C8B	120.7 (19)	S5—C5—C10	122.2 (5)
C7A—C8A—C9A	117.0 (12)	C5—C6—C7	121.6 (6)
C7B—C8B—C9B	115.4 (19)	I7—C7—C8	119.9 (4)
C8A—C9A—C10A	124.0 (13)	I7—C7—C6	120.1 (5)
N1A—C9A—C10A	115.8 (12)	C6—C7—C8	120.0 (6)
N1A—C9A—C8A	120.2 (11)	O8—C8—C9	120.4 (5)
N1B—C9B—C8B	119.4 (17)	O8—C8—C7	120.2 (5)
C8B—C9B—C10B	126 (2)	C7—C8—C9	119.5 (6)
N1B—C9B—C10B	115 (2)	N1—C9—C10	122.8 (6)
C4A—C10A—C9A	119.6 (15)	C8—C9—C10	121.3 (6)
C4A—C10A—C5A	126.7 (16)	N1—C9—C8	115.8 (6)
C5A—C10A—C9A	113.3 (14)	C4—C10—C9	117.0 (6)
C5B—C10B—C9B	118 (2)	C4—C10—C5	126.1 (6)
C4B—C10B—C5B	116 (2)	C5—C10—C9	116.9 (6)
C4B—C10B—C9B	126 (2)	N1—C2—H2	118.00
C3A—C2A—H2A	120.00	C3—C2—H2	118.00
N1A—C2A—H2A	120.00	C4—C3—H3	121.00
C3B—C2B—H2B	117.00	C2—C3—H3	120.00
N1B—C2B—H2B	118.00	C3—C4—H4	120.00
C2A—C3A—H3A	120.00	C10—C4—H4	120.00
C4A—C3A—H3A	120.00	C5—C6—H6	119.00
C2B—C3B—H3B	122.00	C7—C6—H6	119.00
			
O53—S5—C5—C6	−130.4 (5)	C8A—C9A—C10A—C4A	177.6 (14)
O53—S5—C5—C10	51.8 (5)	N1—C2—C3—C4	−0.9 (10)
O52—S5—C5—C6	110.4 (5)	C2—C3—C4—C10	−0.1 (9)
O51—S5—C5—C6	−11.0 (5)	C3—C4—C10—C5	−179.3 (6)
O51—S5—C5—C10	171.1 (5)	C3—C4—C10—C9	0.5 (9)
O52—S5—C5—C10	−67.4 (5)	S5—C5—C6—C7	−177.8 (5)
C2A—N1A—C9A—C10A	3.5 (18)	C10—C5—C6—C7	0.0 (9)
C9A—N1A—C2A—C3A	−1.6 (17)	S5—C5—C10—C4	−1.5 (8)
C2A—N1A—C9A—C8A	−179.2 (11)	S5—C5—C10—C9	178.7 (4)
C9—N1—C2—C3	1.4 (9)	C6—C5—C10—C4	−179.2 (6)
C2—N1—C9—C8	−179.6 (5)	C6—C5—C10—C9	1.0 (8)
C2—N1—C9—C10	−0.9 (9)	C5—C6—C7—I7	177.1 (4)
N1A—C2A—C3A—C4A	1.3 (17)	C5—C6—C7—C8	−0.5 (9)
C2A—C3A—C4A—C10A	−3 (2)	I7—C7—C8—O8	2.6 (7)
C3A—C4A—C10A—C5A	178.0 (15)	I7—C7—C8—C9	−177.6 (4)
C3A—C4A—C10A—C9A	5 (2)	C6—C7—C8—O8	−179.9 (5)
C6A—C5A—C10A—C4A	−176.6 (16)	C6—C7—C8—C9	0.0 (8)
C6A—C5A—C10A—C9A	−4 (2)	O8—C8—C9—N1	−0.4 (8)
C10A—C5A—C6A—C7A	1 (2)	O8—C8—C9—C10	−179.1 (5)
C5A—C6A—C7A—C8A	0.7 (19)	C7—C8—C9—N1	179.8 (5)
C6A—C7A—C8A—C9A	−0.3 (19)	C7—C8—C9—C10	1.1 (9)
C7A—C8A—C9A—C10A	−2 (2)	N1—C9—C10—C4	0.0 (9)
C7A—C8A—C9A—N1A	−179.1 (11)	N1—C9—C10—C5	179.8 (5)
N1A—C9A—C10A—C4A	−5 (2)	C8—C9—C10—C4	178.6 (6)
C8A—C9A—C10A—C5A	4 (2)	C8—C9—C10—C5	−1.5 (8)
N1A—C9A—C10A—C5A	−179.0 (13)		

### Hydrogen-bond geometry (Å, º)

**Table:**

*D*—H···*A*	*D*—H	H···*A*	*D*···*A*	*D*—H···*A*
N1*A*—H1*A*···O53^i^	0.86	1.97	2.783 (10)	157
N1*B*—H1*B*···O53^i^	0.86	1.88	2.725 (16)	166
O8—H8···N1	0.81	2.23	2.693 (7)	117
O8—H8···O52^ii^	0.81	2.13	2.769 (7)	135
O1*W*—H11*W*···O52	0.89	2.18	3.066 (9)	179
O1*W*—H12*W*···O51^iii^	0.90	2.18	3.080 (8)	178
C4—H4···O53	0.93	2.55	3.110 (8)	119
C6—H6···O51	0.93	2.39	2.827 (8)	108
C8*A*—H8*A*···O53^i^	0.93	2.58	3.251 (14)	130

Symmetry codes: (i) −*x*+1, −*y*+1, *z*+1/2; (ii) −*x*+1, −*y*+2, *z*+1/2; (iii) *x*, *y*+1, *z*.

## References

[bb1] Agilent (2012). *CrysAlis PRO* Agilent Technologies, Yarnton, England.

[bb2] Altomare, A., Cascarano, G., Giacovazzo, C. & Guagliardi, A. (1993). *J. Appl. Cryst.* **26**, 343–350.

[bb3] Balasubramanian, T. & Muthiah, P. T. (1996). *Acta Cryst.* C**52**, 2072–2073. 10.1107/s010827019501170x 8624235

[bb4] Farrugia, L. J. (2012). *J. Appl. Cryst.* **45**, 849–854.

[bb5] Flack, H. D. (1983). *Acta Cryst.* A**39**, 876–881.

[bb6] Hemamalini, M., Mu­thiah, P. T., Bocelli, G. & Cantoni, A. (2004). *Acta Cryst.* C**60**, o284–o286. 10.1107/S0108270104001295 15071237

[bb7] Sheldrick, G. M. (2008). *Acta Cryst.* A**64**, 112–122. 10.1107/S0108767307043930 18156677

[bb8] Smith, G., Wermuth, U. D. & Healy, P. C. (2004). *Acta Cryst.* C**60**, o600–o603. 10.1107/S0108270104015057 15295198

[bb9] Smith, G., Wermuth, U. D. & Healy, P. C. (2007). *Acta Cryst.* C**63**, o405–o407. 10.1107/S0108270107025772 17609572

[bb10] Spek, A. L. (2009). *Acta Cryst.* D**65**, 148–155. 10.1107/S090744490804362X PMC2631630 19171970

[bb11] Vogel, A. I. (1964). *Textbook of Macro and Semi-Micro Qualitative Inorganic Analysis*, 4th ed., p. 266. London: Longmans.

